# Acyl-CoA Synthetase 5 Promotes the Growth and Invasion of Colorectal Cancer Cells

**DOI:** 10.1155/2017/7615736

**Published:** 2017-07-20

**Authors:** Shihua Ding, Shaohui Tang, Min Wang, Donghai Wu, Haijian Guo

**Affiliations:** ^1^Department of Gastroenterology, The First Affiliated Hospital, Shenzhen University, Shenzhen 518035, China; ^2^Department of Gastroenterology, The First Affiliated Hospital, Jinan University, Guangzhou 510632, China

## Abstract

**Background and Aims:**

Acyl-CoA synthetase 5 (ACS5) has been reported to be associated with the development of various cancers, but the role of it in colorectal cancer (CRC) is not well understood. The present study aimed to explore the potential role of ACS5 in the development and progression of CRC.

**Methods:**

ACS5 expression in CRC tissues and CRC cell lines was examined, and its clinical significance was analyzed. The role of ACS5 in cell proliferation, apoptosis, and invasion was examined in vitro.

**Results:**

We found that ACS5 expression was upregulated in CRC cells and CRC tissues and that high ACS5 expression was more frequent in CRC patients with excess muscular layer and with poor tumor differentiation. Furthermore, knockdown of ACS5 in HT29 and SW480 cells significantly dampened cell proliferation, induced cell apoptosis, and reduced cell migration and invasion. In contrast, the ectopic overexpression of ACS5 in LOVO and SW620 cells remarkably promoted cell proliferation, inhibited cell apoptosis, and enhanced cell migration and invasion. Enhanced cell growth and invasion ability mediated by the gain of ACS5 expression were associated with downregulation of caspase-3 and E-cadherin and upregulation of survivin and CD44.

**Conclusions:**

Our data demonstrate that ACS5 can promote the growth and invasion of CRC cells and provide a potential target for CRC gene therapy.

## 1. Introduction

Colorectal cancer (CRC) is the third most common cancer and the fourth most common cancer cause of death in the world, accounting for roughly 1.23 million new cases and 608,000 cases of deaths every year [1]. CRC has been closely related to the following risk factors: age, male sex, smoking, family history of colorectal cancer, inflammatory bowel disease, excessive alcohol consumption, high consumption of processed and red meat, obesity, and diabetes [2].

Acyl-CoA synthetase 5 (ACS5) gene encodes an enzyme involved in fatty acid degradation and lipid biosynthesis [3]. Differential expression of ACS5 has been observed in many types of tumors [4–8]. For instance, ACS5 upregulation was related to malignant glioma, but ACS5 was found to be downregulated in small intestine carcinoma [4, 5]. However, the clinical significance and function of ACS5 in CRC are unclear.

In this study, we investigated the expression of ACS5 in CRC tissues and cell lines using immunohistochemistry, quantitative real-time polymerase chain reaction (qRT-PCR), and western blotting. In addition, we identified the correlations between ACS5 expression levels and clinicopathological features in CRC patients. Furthermore, we explored the functional role of ACS5 in CRC cells proliferation, apoptosis, and invasion by in vitro experiments.

## 2. Materials and Methods

### 2.1. Cell Culture

Five CRC cell lines (HCT116, HT29, LOVO, SW620, and SW480), which were obtained from American Type Culture Collection (Manassas, VA, USA), were grown in Dulbecco's modified Eagle medium (Gibco BRL, Rockville, MD, USA) containing 10% fetal bovine serum (Gibco BRL) and 100 U/ml penicillin/streptomycin at 37°C under 5% CO_2_.

### 2.2. Immunohistochemistry and Scoring

Immunohistochemistry (IHC) of tissue specimens was treated in routinely processed, formalin-fixed, paraffin-embedded sections using a streptavidin-biotin complex method. The specimens were autoclaved for 10 min and then were incubated with anti-ACS5 antibody overnight. The specimens were washed and incubated with secondary antibodies at 37°C for 2 h. Detection was carried out using 3′,3-diaminobenzidine tetrahydrochloride (DAB). Finally, specimens were counterstained with haematoxylin.

IHC analysis was performed as described elsewhere [9]. Briefly, five fields were randomly selected, and three slides for each specimen were calculated. The intensity of the staining fell into categories of 0 (no staining), 1 (weak staining), 2 (moderate staining), and 3 (strong staining), respectively. The staining extent was graded from 0 to 3, according to the percentage of positive cells (0: <10%; 1: 10%–25%; 2: 25%–50%; and 3: >50%). The total ACS5 immunostaining score was calculated using staining intensity × the percentage of positive cells score, ranging between 0 and 9. Samples with the total score of ≥1 were defined as high ACS5 expressers, and samples with the total score of 0 were considered as low ACS5 expressers. For the negative control, PBS was used instead of primary antibody. When there were divergences between the two pathologists in their scoring, an average score was used.

### 2.3. RNA Interference and Transfection

The siRNA targeting human ACS5 (NCBI database NM_016234) was as follows: 5′-GCAAUUACGUGAAGCUGGA-3′. A control siRNA oligonucleotide, which does not match any known human coding cDNA, was used as the negative control. All siRNAs were purchased from Sigma (Deisenhofen, Germany). siRNAs were introduced into the HT29 and SW480 cells with Lipofectamine™ RNAiMAX (Invitrogen, Carlsbad, CA, USA), according to the manufacturer's instructions. The cells were divided into 3 groups: the blank control group (the untreated cells), the negative control group (cells infected with nonsilencing siRNA), and the ACS5 siRNA group (cells infected with ACS5 siRNA). We employed real-time RT-PCR and western blot to evaluate the inhibitory effects.

### 2.4. Plasmid Construction and Transfection

The vector pcDNA3.1(+) (Invitrogen), containing a human cytomegalovirus immediate-early (CMV) promoter, was designed for high-level, constitutive expression in a variety of mammalian cell lines. ACS5 cDNAs were amplified by PCR method using the following primers: 5′-cgcggatccgccaccATGGACGCTCTGAAGCCACCC-3′ (BamHI restriction site) and 5′-ccgctcgagCTAATCCTGGATGTGCTCATACAGG-3′ (XhoI restriction site). The ACS5 overexpression vector (pcDNA3.1(+)-ACS5) was created by cloning the ACS5 coding sequence into the BamHI/XhoI site of pcDNA3.1(+). All constructed vectors were confirmed by DNA sequencing. The cells were divided into 3 groups: the blank control group (the untreated cells), the negative control group (transfected with empty vector pcDNA3.1(+)), and the ACS5 overexpression group (transfected with pcDNA3.1(+)-ACS5). LOVO and SW620 cells transfection were performed using Lipofectamine 2000 (Invitrogen), according to the manufacturer's instructions. Overexpression efficiency was measured with real-time RT-PCR and western blot.

### 2.5. Quantitative RT-PCR

Total mRNA was extracted from cultured CRC cells and then reversely transcribed into cDNA using 10 units of Reverse Transcriptase XL (AMV) (TaKaRa, Kyoto, Japan) according to the manufacturer's recommendations. Real-time quantitative RT-PCR was carried out using SYBR Green qPCR SuperMix (Invitrogen). A cycle threshold (CT) was assigned at the beginning of the logarithmic phase of PCR amplification. The relative gene expression was calculated using the 2^−ΔΔCT^ method. The fragment of ACS5 was amplified using the following primers: upstream, 5′-GGAAGGGTTCGTGTAATTGT-3′; and downstream, 5′-CCAGTCCCCAGGTAATGTAA-3′. We selected 18 srRNA as an internal loading control for quantitative RT-PCR. All tests were repeated three times.

### 2.6. Western Blot Analysis

Cells were lysed with RIPA Lysis Buffer (Thermo Fisher Scientific, Waltham, MA, USA) 48 h after transfection. Supernatants were collected, and protein was measured using the BCA Assay Kit (Thermo Fisher Scientific). Protein samples were subjected to SDS-PAGE followed by transfer of protein to nitrocellulose membranes (Bio-Rad, Hercules, CA, USA). We incubated membranes for 1 h with an appropriate dilution of the primary antibodies against ACS5 (Santa Cruz Biotechnology, Santa Cruz, USA) followed by incubation with the horseradish peroxidase-conjugated second-step antibody (Santa Cruz Biotechnology) and visualized them using an enhanced chemiluminescence detection system (Amersham Biosciences, Piscataway, NJ, USA). A GAPDH (Santa Cruz Biotechnology) was used as an internal control. Each experiment was repeated thrice and all reactions were carried out in triplicate.

### 2.7. Cell Proliferation Assay

The cell proliferation was evaluated using the 3-(4,5-dimethylthiazol-2-yl)-5-(3-carboxymethoxyphenyl)-2-(4-sulfophenyl)-2H-tetrazolium (MTS) method. In brief, we used an MTS kit (cellTiter 96 AQ, Promega, Madison, WI). At the indicated time points (0 h, 24 h, 48 h, and 72 h), cells (100 *μ*L/well in 96-well plates) were treated with 10 *μ*L of MTS in phenazine ethosulfate solution, and the mixture was incubated. Then, we recorded the absorbance at 490 nm. Three independent experiments were performed for each cell line assayed.

### 2.8. Cell Apoptosis Analysis

Following culture for 48 h, the cells were harvested, washed with PBS, resuspended in the binding buffer, and incubated for 15 min in the dark with propidium iodide (PI) and Annexin V/fluorescein isothiocyanate (FITC). The stained cells were immediately analyzed by flow cytometry (Beckman Coulter, Brea, CA, USA). The experiment was independently repeated three times.

### 2.9. Transwell Migration and Invasion Assay

The transwells (Costar, USA) with 8 *μ*m pore polycarbonate membranes (Corning, NY, USA) were left uncoated or were coated with Matrigel (BD Biosciences, Franklin Lakes, NJ, USA) before use in migration assays and invasion assays, respectively. Matrigel was added to each transwell upper chamber and placed in a 37°C incubator for 2 h to solidify. Cells were seeded in the transwell upper chamber. The transwell assays were performed according to the manufacturer's instructions. After transwell chambers were incubated for 24 h at 37°C in a humidified incubator with 5% CO_2_, the lower chamber was stained with crystal violet. Cell migration/invasion was evaluated by counting the cells that had migrated/invaded into the filters. Each assay was performed in triplicate and repeated three times.

### 2.10. Statistical Analysis

The statistical software package SPSS 16.0 (SPSS, Inc., Chicago, IL) was applied. Quantitative data were evaluated by independent sample *t*-test followed by Mann-Whitney *U* test. Categorical data were done by the *χ*^2^ or Fisher's exact tests, based on the absolute numbers included in the analysis. A *P* value less than 0.05 was regarded statistically significant.

## 3. Results

### 3.1. Expression of ACS5 in CRC Cell Lines

**Figure 1:**
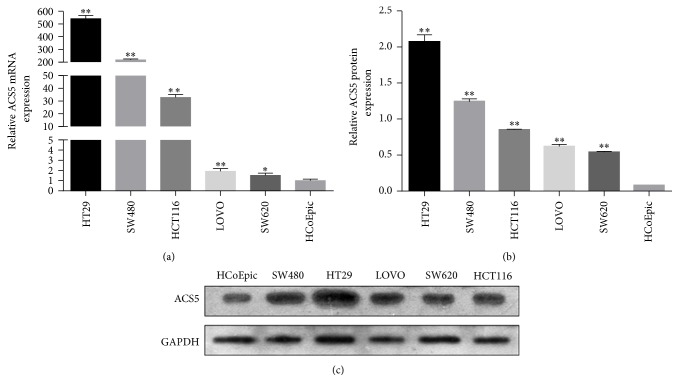
Expression of ACS5 in CRC cell lines. The ACS5 mRNA and protein levels were significantly upregulated in five CRC cells compared with the normal cell line (HCoEpiC). ^*∗∗*^*P* < 0.01, ^*∗*^*P* < 0.05 versus HCoEpiC.

In order to determine whether the expression of ACS5 was different between CRC cell lines and human normal colonic epithelial cell line, quantitative RT-PCR and western blot analysis were carried out. As shown in Figure 1, the ACS5 mRNA and protein levels were higher in CRC cell lines than in human normal colonic epithelial cell line. In the five CRC cell lines, we found two cell lines with relatively low levels of ACS5, LOVO, and SW620 and two cell lines with relatively high amounts of ACS5, HT29, and SW480.

### 3.2. Expression of ACS5 in CRC, Adenoma, and Normal Mucosa Tissues

**Figure 2:**
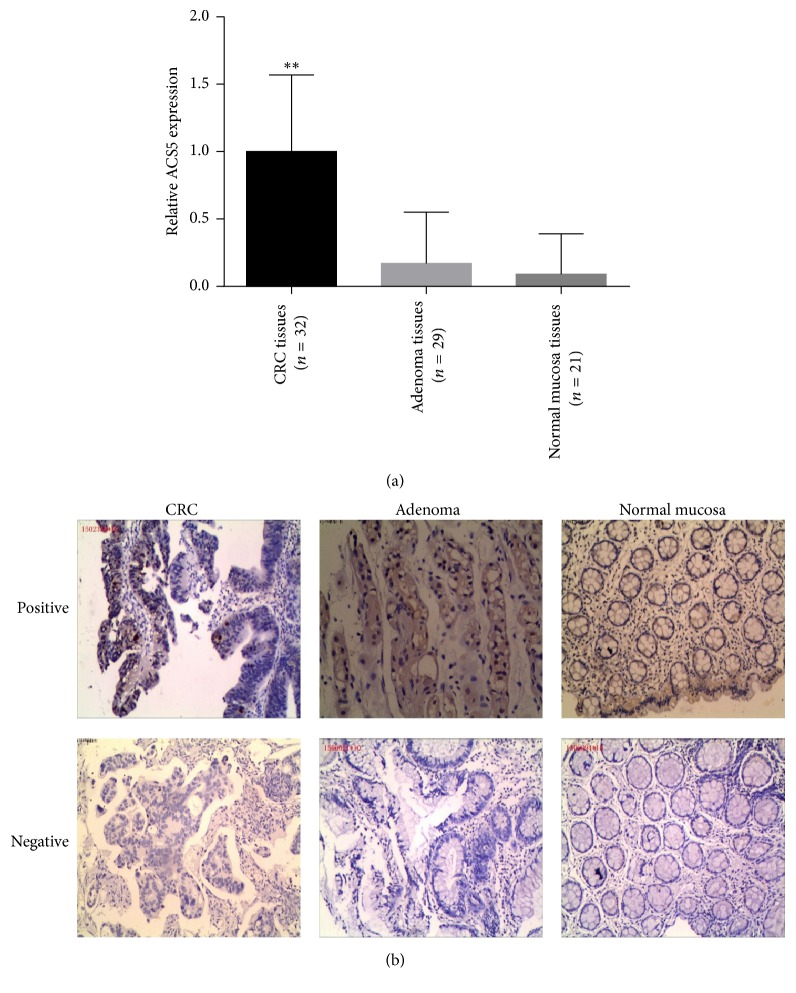
ACS5 overexpression in CRC tissues. (a) The ACS5 protein level was significantly upregulated in CRC tissues compared with adenoma tissues and normal mucosa tissues. (b) Representative photographs of ACS5 staining in CRC, adenoma, and normal mucosa tissues (stain, hematoxylin; magnification, ×100). ^*∗∗*^*P* < 0.01 versus adenoma tissue or normal mucosa tissue.

To investigate the role of ACS5 in CRC, we examined the level of ACS5 in 32 human CRC tissues, 29 adenoma tissues, and 21 normal mucosa tissues (obtained from the Institute of Gastrointestinal Surgery) by immunohistochemistry (IHC) analysis. Typical immunostaining of ACS5 expression in CRC and control tissues is presented in Figure 2(b). The result revealed that the IHC scores of ACS5 protein expression were significantly increased in the CRC tissues compared with normal mucosa and adenoma tissues, and there was no statistical difference in the IHC scores of ACS5 protein expression between adenoma tissues and normal mucosa tissues (Figure 2(a)). The CRC patients with the high IHC ACS5 scores were more often associated with poor tumor differentiation and excess muscular layer compared with the patients with low IHC ACS5 scores, whereas there was no difference between the high and low IHC ACS5 score groups regarding age, sex, lymph node metastasis, and Duke's stage (Table 1). The above results suggest that ACS5 overexpression is involved in CRC development and is closely correlated to poor tumor differentiation and excess muscular layer in patients with CRC.

### 3.3. Effect of ACS5 on the Proliferation of CRC Cells

**Figure 3:**
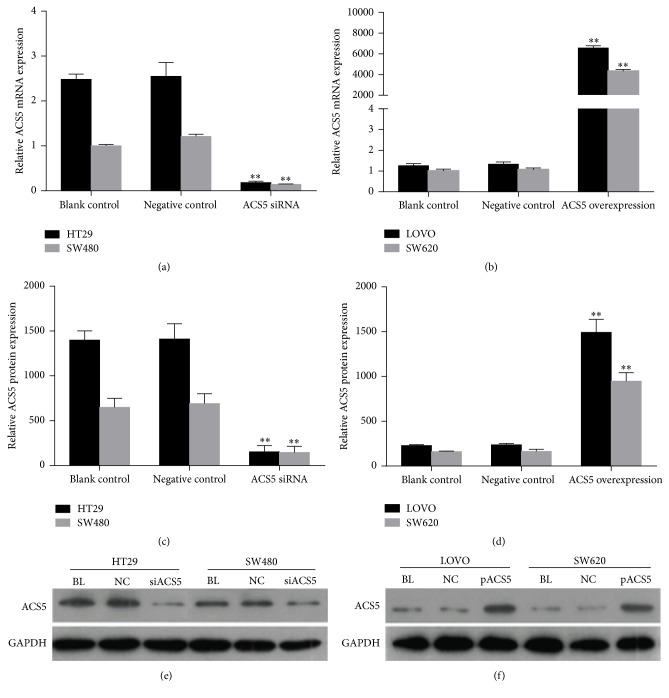
The level of ACS5 expression in CRC cells after transfection. ((a), (c)) ACS5 mRNA and protein levels were significantly lower in HT29 and SW480 cells with ACS5 knockdown than those in the negative control or blank control. ((b), (d)) ACS5 mRNA and protein levels were remarkably upregulated in LOVO and SW620 cells with ACS5 overexpression compared with the negative control or blank control. ((e), (f)) Representative western blot results of ACS5 protein expression. ^*∗∗*^*P* < 0.01 versus negative control or blank control group. BL, blank control; NC, negative control; siACS5, ACS5 siRNA; pACS5, pcDNA3.1(+)-ACS5.

**Figure 4:**
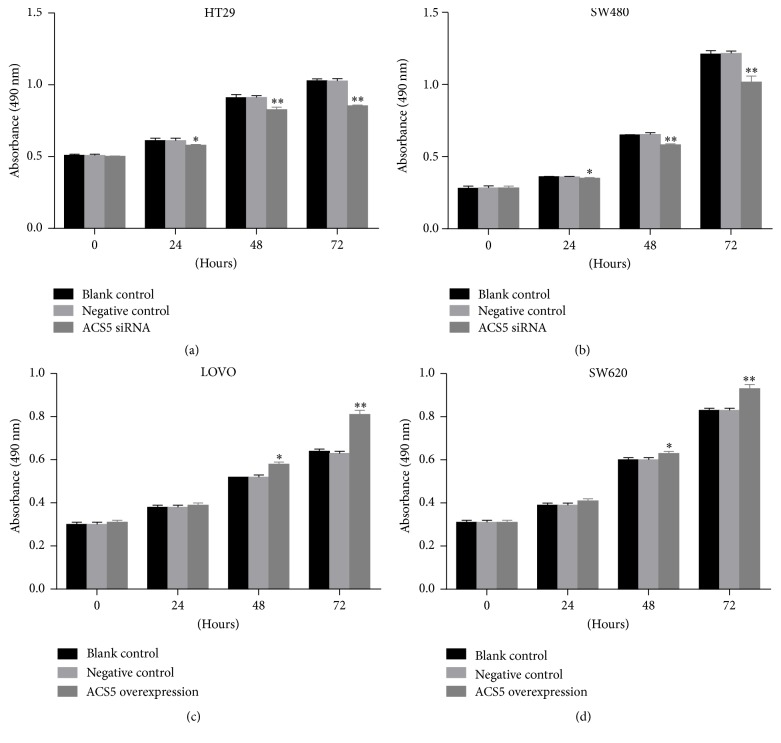
Effect of ACS5 on the proliferation of CRC cells. Cell proliferation was evaluated by MTS, and the results of cell growth were expressed by absorbance at 490 nm. ((a), (b)) The proliferation inhibition of HT29 and SW480 cells with ACS5 knockdown was observed compared with negative control and blank control at 24 h, 48 h, and 72 h. ((c), (d)) The proliferation promotion of LOVO and SW620 cells with ACS5 overexpression was observed compared with negative control and blank control at 48 h and 72 h. ^*∗∗*^*P* < 0.01, ^*∗*^*P* < 0.05 versus negative control or blank control group.

As shown in Figure 3, we silenced ACS5 in HT29 and SW480 cells with high levels of endogenous ACS5, and we utilised a plasmid to upregulate ACS5 expression in the LOVO and SW620 cells with low endogenous ACS5 levels. To determine the effect of ACS5 on the proliferation of CRC cells, we used a MTS assay at different time points (0 h, 24 h, 48 h, and 72 h, resp.). As shown in Figures 4(a) and 4(b), the proliferation of the HT29 and SW480 cells in the knockdown of ACS5 group was observably lower than that of the cells treated with negative control, or the untreated cells (blank control). In contrast, the proliferation of the LOVO and SW620 cells in the ectopic overexpression of ACS5 group was significantly higher than that of the cells treated with negative control, or the untreated cells (blank control) (Figures 4(c) and 4(d)). This finding suggests that ACS5 plays an important role in CRC cell proliferation.

### 3.4. Effect of ACS5 on the Apoptosis of CRC Cells

**Figure 5:**
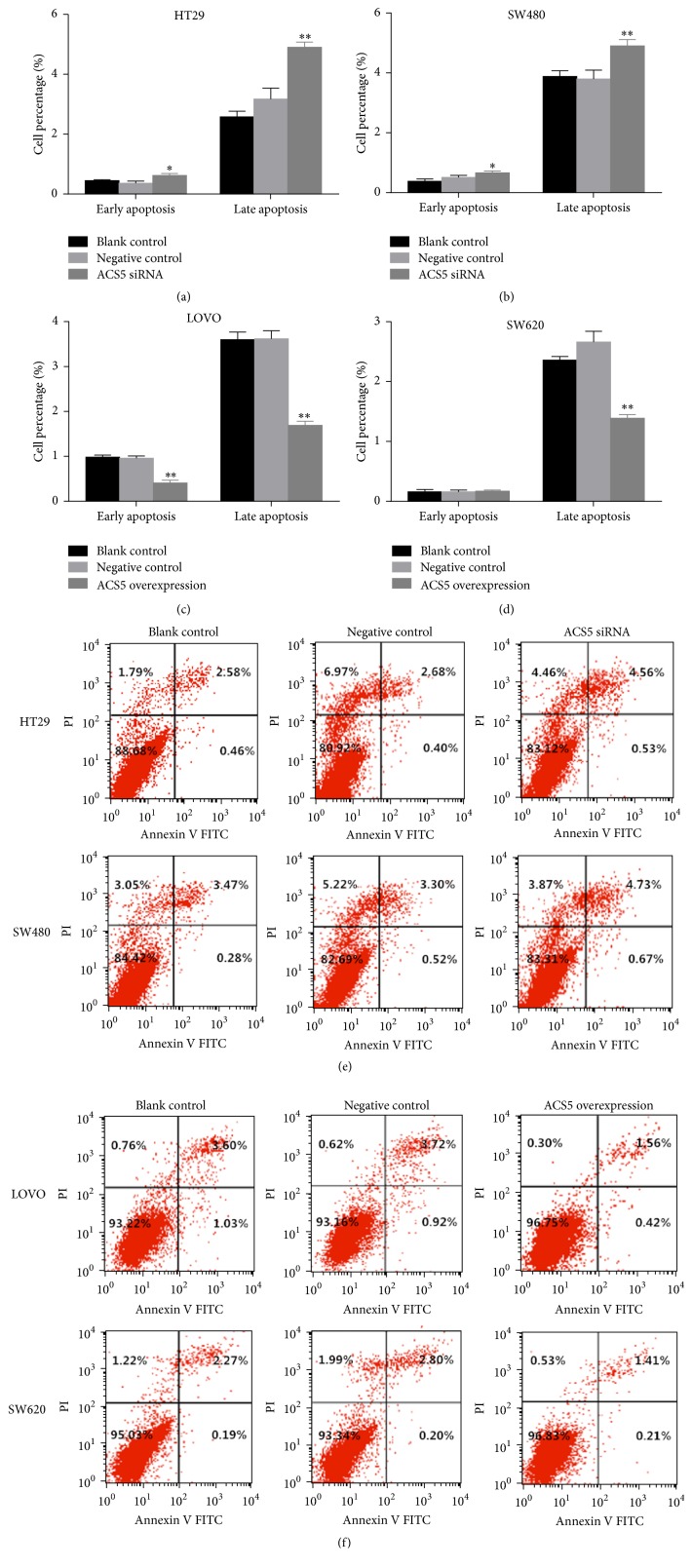
Effect of ACS5 on the apoptosis of CRC cells. ((a), (b), (e)) The percentage of early and late apoptotic cells was higher in HT29 and SW480 cells with ACS5 knockdown than that in negative control or blank control cells. ((c), (d), (f)) The percentage of late apoptotic cells was lower in LOVO and SW620 cells with ACS5 overexpression than that in negative control or blank control cells. ^*∗*^*P* < 0.05, ^*∗∗*^*P* < 0.01 versus negative control or blank control group.

The effect of ACS5 on CRC cell apoptosis was analyzed using a flow cytometer. Silencing of ACS5 remarkably increased the percentage of early and late apoptotic cells in both HT29 (Figures 5(a) and 5(e)) and SW480 (Figures 5(b) and 5(e)) cells compared with the cells treated with negative control, or the untreated cells (blank control). In contrast, the ectopic overexpression of ACS5 significantly decreased the percentage of early and late apoptotic cells in LOVO (Figures 5(c) and 5(f)) cells and remarkably reduced the percentage of late apoptotic cells in SW620 (Figures 5(d) and 5(f)) cells compared with the cells treated with negative control, or the untreated cells (blank control). This outcome indicates that ACS5 can inhibit the apoptosis of the CRC cells.

### 3.5. Effect of ACS5 on the Migration and Invasion of CRC Cells

**Figure 6:**
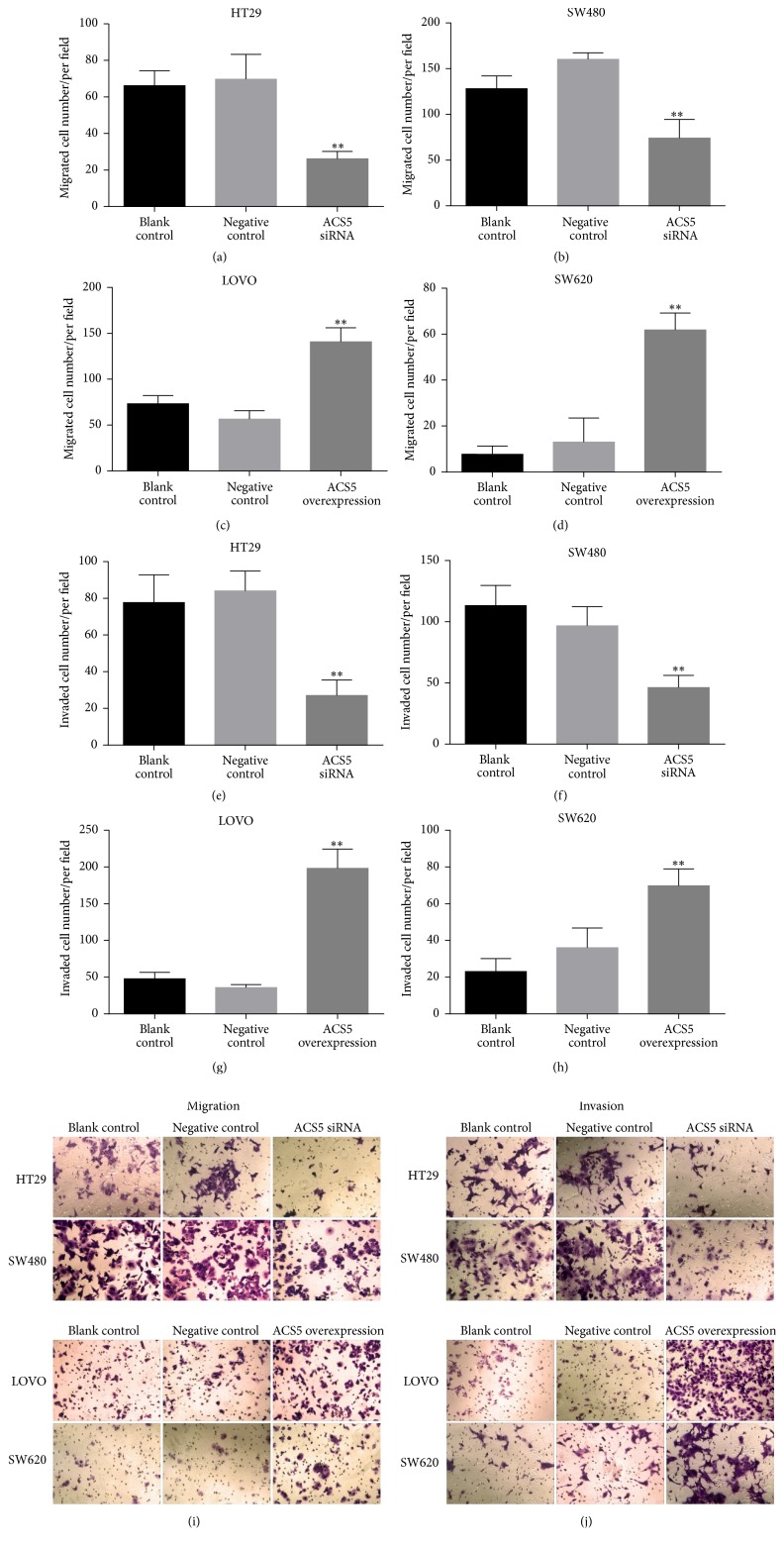
Effect of ACS5 on the migration and invasion of CRC cells. ((a)–(d), (i)) Analysis of migration capability of CRC cells. ((e)–(h), (j)) Analysis of invasion capability of CRC cells. Both the migrated and invaded number of HT29 and SW480 cells with ACS5 knockdown were significantly decreased compared with negative control or blank control. Inversely, both the migrated and invaded number of LOVO and SW620 cells with ACS5 overexpression were remarkably increased compared with negative control or blank control. ^*∗∗*^*P* < 0.01 versus negative control or blank control group.

To further evaluate the effect of ACS5 on the migration and invasion capability of CRC cells, the transwell assays were performed. Knockdown of ACS5 remarkably decreased the migrated and invaded number of both HT29 (Figures 6(a), 6(e), 6(i), and 6(j)) and SW480 (Figures 6(b), 6(f), 6(i), and 6(j)) cells compared with the cells treated with negative control, or the untreated cells (blank control). Inversely, the ectopic overexpression of ACS5 significantly increased the migrated and invaded number of both LOVO (Figures 6(c), 6(g), 6(i), and 6(j)) and SW620 (Figures 6(d), 6(h), 6(i), and 6(j)) cells compared with the cells treated with negative control, or the untreated cells (blank control). These results indicate that ACS5 can promote the migration and invasion capability of the CRC cells.

### 3.6. ACS5 Affects CRC Cells Growth and Metastasis through the Modulation of Survivin and CD44 Expression

**Figure 7:**
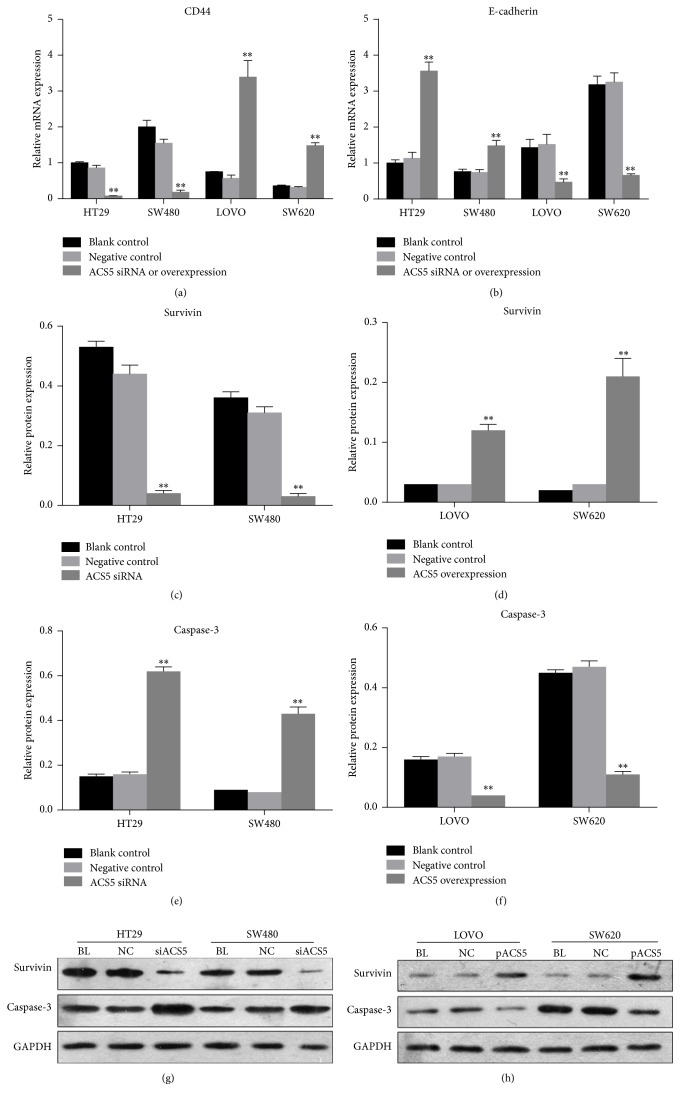
ACS5 affects CRC cells growth and metastasis through the modulation of survivin and CD44 expression. ((a), (b), (c), (e), (g)) The downregulation of ACS5 in HT29 and SW480 cells resulted in a significant reduction in survivin and CD44 expression and striking increase in caspase-3 and E-cadherin expression. ((a), (b), (d), (f), (h)) The expression of survivin and CD44 was significantly elevated, while the levels of caspase-3 and E-cadherin expression were markedly decreased in ACS5 overexpressed SW620 and LOVO cells. ^*∗∗*^*P* < 0.01 versus negative control or blank control group. BL, blank control; NC, negative control; siACS5, ACS5 siRNA; pACS5, pcDNA3.1(+)-ACS5.

To investigate the potential mechanism by which ACS5 affects cellular proliferation and invasion, we explored the change in cell apoptosis and invasion-related molecules in these transfected cells. The downregulation of ACS5 in HT29 and SW480 cells resulted in a significant reduction in survivin and CD44 expression and striking increase in caspase-3 and E-cadherin expression (Figures 7(a)–7(c), 7(e), and 7(g)). Additionally, the expression of survivin and CD44 was significantly elevated, while the levels of caspase-3 and E-cadherin expression were markedly decreased in ACS5 overexpressed SW620 and LOVO cells (Figures 7(a), 7(b), 7(d), 7(f), and 7(h)).

## 4. Discussion

Colorectal cancer is notorious for its high incidence and poor prognosis [10, 11]. Although comprehensive treatments are available that include radiotherapy, surgery, and chemotherapy, the therapeutic efficacy is not optimistic [12, 13]. However, one of the major obstacles in improving the quality of life and survival rate is the high frequency of recurrence and metastasis [14, 15]. Therefore, it is imperative to discover novel therapeutic targets and reliable prognostic biomarkers to improve the management of CRC patients.

ACS5 gene is located on chromosome 10q25.1-q25.2, spans approximately 46 kb, comprises 21 exons and 22 introns, and encodes a 683-amino acid protein [4]. As a member of the ACS family, ACS5 not only produces acyl-CoA for numerous metabolic pathways including cellular lipid metabolism, transcriptional regulation, intracellular protein transportation, protein acylation, and protein kinase C mediated signal transduction, but also facilitates fatty acid transportation into cells [4, 16, 17]. ACS5 is predominantly detected in the normal liver, small intestine, white preadipocytes, and brown adipose tissue [18, 19]. In recent years, it has been reported that ACS5 is involved in cancer progression [20]. However, whether the role of ACS5 is being a tumor suppressor or a tumor promoter remains controversial, and its underlying mechanisms have not been clarified. On one hand, ACS5 is suggested as a tumor promoter in malignant glioma [21]. On the other hand, ACS5 is considered to be a tumor suppressor in hepatocellular carcinoma [8]. The above researches suggest that ACS5 may be able to act as both a tumor suppressor and a tumor promoter depending on physiopathology conditions, tissue specificity, and other factors. At the present time, we have limited understanding about the in vitro role of ACS5 in the development and progression of CRC.

In the current study, we first used quantitative RT-PCR and western blot analysis to detect the expression of ACS5 mRNA and protein levels in CRC cell lines (HT29, SW480, LOVO, SW620, and HCT116) and human normal colonic epithelial cell line (HCoEpiC). The results showed that the ACS5 mRNA and protein levels were higher in CRC cell lines than in human normal colonic epithelial cell line, suggesting that ACS5 overexpression may be associated with the development of CRC at the cellular level.

On the other hand, we revealed that the IHC scores of ACS5 protein in CRC tissues were significantly higher than those in adenoma tissues and normal mucosa tissues. There was no significant difference in the scores between adenoma tissues and normal mucosa tissues. Additionally, clinicopathological data showed that high ACS5 expression was more frequent in CRC patients with excess muscular layer and with poor tumor differentiation. These findings suggest the possibility that ACS5 overexpression may play an important role in the development of CRC and may be linked with tumor differentiation and invasion. In a previous study, Gassler et al. measured ACS5 expression in 15 CRC patients using RT-PCR and western blot. They revealed that ACS5 expression level was significantly upregulated in adenoma tissues and CRC tissues compared with normal mucosa tissues and that the expression of ACS5 expression level was also higher in adenoma tissues than that in CRC tissues [6]. The reasons for this difference from our study were not clear and needed further exploration. In addition, they failed to employ in vitro and in vivo experiments to further explore the role of ACS5 in the progression of CRC. Similar to our study, Gassler et al. showed that the higher the expression level of ACS5 in endometrial carcinoma, the higher the degree of tumor differentiation [22]; Gaisa et al. reported that the expression level of ACS5 in normal urothelial tissue was significantly higher than that in tumor tissue and reported a gradual loss of ACS5 expression with decreasing cellular differentiation in urothelial cancers [23]. These two studies suggested that ACS5 was a tumor suppressor, which was contrary to our findings in colorectal cancer, that may be related to the type of tumor.

Next, we silenced ACS5 in HT29 and SW480 cells with high levels of endogenous ACS5, and we utilised a plasmid to upregulate ACS5 expression in the LOVO and SW620 cells with low endogenous ACS5 levels. The quantitative RT-PCR and western blot data showed that ACS5 gene expression was successfully silenced in HT29 and SW480 cells and overexpressed in LOVO and SW620 cells.

Then, we employed in vitro experiments to further explore the role of ACS5 in the development and progression of CRC. Knockdown of ACS5 expression led to decreased proliferation, apoptosis induction, and decreased migration and invasion capability of HT29 and SW480 cells. In contrast, the ectopic overexpression of ACS5 resulted in increased proliferation, apoptosis inhibition, and increased migration and invasion of LOVO and SW620 cells. Similarly, Mashima et al. demonstrated that the stable expression of ACS5 strongly inhibited brain cancer cell death [24]. Moreover, Mashima et al. also reported that in vivo treatment with ACS5 siRNA significantly suppressed the growth of tumor in glioma [21]. These data, together with our findings, indicate that ACS5 may play an import role in promoting tumor growth and metastasis in CRC.

The molecular mechanisms by which ACS5 promotes cancer cell proliferation and metastasis remain unclear. Some data argue for an involvement of LKB1/AMPK/mTOR signal transduction pathway, whereas others indicate ACS5-related changes in cytochrome C release from mitochondria as apoptosis-relevant mechanisms. The serine/threonine kinase LKB1 is a master kinase involved in cellular responses such as energy metabolism, cell polarity, and cell growth. LKB1 regulates these crucial cellular responses mainly via AMPK/mTOR signaling. Various cancers have been associated with impaired AMPK activation and/or mTOR inhibition [25]. Klaus et al. reported that the tumor promoter role of ACS5 was possibly associated with the regulation of p53 pathways, which related to numerous cellular processes important for cell growth and survival [26]. LKB1 is also reportedly a mediator of p53-dependent apoptosis [27]. Thus, ACS5 may inhibit cell apoptosis by modulating the LKB1/AMPK/mTOR signal transduction pathway, though the role of p53 in these processes remains unclear. As demonstrated in previous studies, Acyl-CoA, the primary product of ACS5 activity, is able to affect several mitochondrial proteins, including adenine nucleotide translocase, which is potentially involved in cytochrome C release from mitochondria during apoptosis [28–30]. A probable pathway to mediate ACS5 antiapoptotic activities involves the diminishing of free fatty acids, which have been reported to promote the opening of the mitochondrial permeability transition pore or to cause cytochrome C release [31, 32]. The diminution of free fatty acids could be induced by ACS5 activity [33]. In the present study, we examined the expression of the cell apoptosis-related protein caspase-3 and survivin [34, 35]. We found that ACS5 overexpression could increase survivin protein (antiapoptotic protein) levels and decrease caspase-3 protein (proapoptotic protein) levels. Accumulating evidence shows that the genes E-cadherin and CD44 are involved in tumor metastasis [36, 37]. Here we found that inhibition of ACS5 expression significantly reduced CD44 mRNA levels but increased E-cadherin mRNA levels. This suggests that ACS5 may promote the metastasis and invasion of CRC through upregulation of survivin and CD44 expression.

In conclusion, our study has demonstrated that ACS5 expression was increased in CRC cells and CRC tissues and its upregulation closely correlated to poor tumor differentiation and excess muscular layer in patients with CRC. In addition, we verified that ACS5 can promote CRC cells growth and invasion in vitro, possibly through modulating survivin and CD44 expression. Our findings provide useful information for understanding the role of ACS5 in the development and progression of CRC and as a potential target for CRC gene therapy.

## Figures and Tables

**Table 1:** Relationship between ACS5 expression and clinical pathological features of CRC *n* (%).

Variables	Cases (32)	ACS5 expression		*P* value
		High (27)	Low (5)	
*Age*				
<60	12	8 (29.6)	4 (80.0)	0.053
≥60	20	19 (70.4)	1 (20.0)	
*Gender*				
Male	19	17 (63.0)	2 (40.0)	0.374
Female	13	10 (37.0)	3 (60.0)	
*Depth of tumor invasion*				
Not exceeding muscular layer	8	4 (14.8)	4 (80.0)	**0.009**
Exceeding muscular layer	24	23 (85.2)	1 (20.0)	
*Lymph node metastasis*				
Negative	18	13 (48.1)	5 (100.0)	0.052
Positive	14	14 (51.9)	0 (0.0)	
*Degree of differentiation*				
Well	8	5 (18.5)	3 (60.0)	
Moderate	10	8 (29.6)	2 (40.0)	**0.041**
Poor	14	14 (51.9)	0 (0.0)	
*Duke's stage*				
A	7	6 (22.2)	1 (20.0)	
B	10	7 (25.9)	3 (60.0)	0.328
C + D	15	14 (51.9)	1 (20.0)	
